# GGT5 facilitates migration and invasion through the induction of epithelial–mesenchymal transformation in gastric cancer

**DOI:** 10.1186/s12920-024-01856-0

**Published:** 2024-04-05

**Authors:** Zhuang Luo, Yong Chen, Bangquan Chen, Ziming Zhao, Rongfan Wu, Jun Ren

**Affiliations:** 1 Department of Proctology, Huai’an Hospital of Traditional Chinese Medicine, Huai’an, 223001 China; 2 Northern Jiangsu People’s Hospital Affiliated to Yangzhou University, Yangzhou, 225001 China; 3 General Surgery Institute of Yangzhou, Yangzhou University, Yangzhou, 225001 China; 4 Department of Hepatobiliary Pancreatic Surgery, Gaochun People’s Hospital of Nanjing, Nanjing, 211300 China

**Keywords:** GGT5, Gastric cancer, EMT, Prognosis, Migration

## Abstract

**Background:**

Gamma-glutamyltransferase 5 (GGT5), one of the two members in the GGT family (GGT1 and GGT5), plays a crucial role in oxidative regulation, inflammation promotion, and drug metabolism. Particularly in the tumorigenesis of various cancers, its significance has been recognized. Nevertheless, GGT5’s role in gastric cancer (GC) remains ambiguous. This study delves into the function and prognostic significance of GGT5 in GC through a series of in vitro experiments.

**Methods:**

Employing online bioinformatics analysis tools such as The Cancer Genome Atlas (TCGA), Gene Expression Omnibus (GEO), Kaplan–Meier plotter, and cBioPortal, we explored GGT5 characteristics and functions in GC. This encompassed aberrant expression, prognostic value, genomic alterations and mutations, immune cell infiltration, and associated signaling pathways. Immunohistochemistry was conducted to assess GGT5 expression in GC and adjacent normal tissues. Subsequently, univariate and multivariate logistic regression analyses were applied to investigate the associations between GGT5 and clinical characteristics. CCK8, wound healing, and migration assays were utilized to evaluate the impact of GGT5 on cell viability and migration. Additionally, Gene Set Enrichment Analysis (GSEA) and Western blot analysis were performed to scrutinize the activity of the epithelial–mesenchymal transformation (EMT) signaling pathway under GGT5 regulation.

**Results:**

GGT5 exhibits upregulation in gastric cancer, with its overexpression significantly linked to histological differentiation in GC patients (*P* < 0.05). Multivariate analysis indicates that elevated GGT5 expression is an independent risk factor associated with poorer overall survival in gastric cancer patients (*P* < 0.05). In vitro experiments reveal that downregulation of GGT5 hampers the proliferation and migration of GC cell lines. Finally, GSEA using TCGA data highlights a significant correlation between GGT5 expression and genes associated with EMT, a finding further confirmed by Western blot analysis.

**Conclusions:**

GGT5 emerges as a promising prognostic biomarker and potential therapeutic target for GC.

**Supplementary Information:**

The online version contains supplementary material available at 10.1186/s12920-024-01856-0.

## Introduction

Gastric Cancer (GC) stands as the fifth most prevalent cancer type and the fourth leading cause of cancer-related mortality worldwide [1]. With the increasing adoption of screening gastroscopy and advancements in medical technology, there has been a reduction in the incidence and death rates associated with gastric cancer [2]. Presently, the primary approach to treating gastric cancer involves surgery or endoscopic resection coupled with standard adjuvant chemotherapy strategies, leading to substantial improvements in overall survival (OS) and disease-free survival (DFS). Nevertheless, the prognosis for advanced GC patients remains unsatisfactory [3]. Recently, there has been promise in the realm of immunotherapy and targeted therapies, including immune inhibitors targeting PD-L1, which demonstrate potential in enhancing the prognosis for individuals with unstable microsatellite cancer [4]. However, the benefits of such immune inhibitors are realized by only a limited population [5]. The introduction of human epidermal growth factor receptor 2 (HER2) inhibitors, such as trastuzumab, pertuzumab, and lapatinib, has contributed to advancements in the outcomes of advanced gastric cancer patients [6]. Despite this progress, the clinical effectiveness of these drugs is hampered by acquired resistance to trastuzumab and the low positive rate of HER2 in GC cases [7]. Therefore, an imperative exists to discover novel potential prognostic biomarkers or therapeutic targets for the diagnosis and targeted therapy of GC patients.

Gamma-glutamyltransferase 5 (GGT5) belongs to the GGT family, which includes GGT1 and GGT5. It exhibits widespread distribution in various tissues, with particularly elevated expression in the liver, kidney, and alveolar macrophages [8]. Operating as a vital liver enzyme associated with the cell membrane, GGT participates in extracellular glutathione metabolism. Its role involves the cleavage of glutathione peptides to maintain the balance of glutathione in the human body [9]. Comprehensive functional analyses have highlighted GGT5’s pivotal involvement in oxidative regulation, inflammatory promotion, drug metabolism, and immune modulation [10, 11]. Furthermore, GGT5 assumes a significant role in the initiation of several tumors. Recent research has unveiled a correlation between elevated GGT5 levels and the development of colon cancer [12]. Wei’s findings suggest that heightened GGT5 levels in cancer-associated fibroblasts (CAFs) contribute to cancer cell survival and drug resistance, indicating GGT5’s potential as a therapeutic target in lung adenocarcinoma [13]. Bioinformatic studies based on clinical gastric cancer samples from the Gene Expression Omnibus (GEO) and The Cancer Genome Atlas (TCGA) databases indicate GGT5’s inclusion in prognostic gene signatures. Furthermore, the overexpression of GGT5 is inversely linked to the survival of gastric cancer patients [14, 15].

The studies mentioned earlier solely examined the prognostic significance of GGT5 in tumors, encompassing GC. Nonetheless, the role of GGT5 in gastric cancer and its specific mechanisms remain unexplored. Previous research, employing bioinformatic analysis, identified GGT5 overexpression in GC compared to normal tissues. Building on this discovery, we conducted additional analyses and experiments to delve into its attributes and underlying mechanisms within the context of GC. This investigation seeks to affirm GGT5’s potential as a novel biomarker and therapeutic target, contributing fresh perspectives on the molecular mechanisms underpinning GC.

## Materials and methods

### Analysis of TCGA and oncomine databases

To forecast GGT5 expression levels in gastric cancer (GC) and normal gastric tissues, we utilized the cancer-related public databases Oncomine, GTEx, and TCGA. In the Oncomine database, we inputted the gene name “GGT5” and opted for the module of differential gene analysis across various datasets to retrieve outcomes. Additionally, the GEPIA browser (http://gepia.cancer-pku.cn/), an online tool for TCGA and GTEx project data analysis, was employed to assess GGT5 expressions between primary GC and normal gastric tissues [16].

### Analysis of genetic variation

Exploration of the genetic mutation and alteration in GGT5, along with its association with the survival of patients with gastric cancer, was undertaken utilizing the cBioPortal database [17]. Furthermore, the online database, The Catalogue of Somatic Mutations, was employed to investigate the distribution of genetic changes in GGT5 [18].

### Gene set enrichment analysis

The Broad Institute website (http://www.broadinstitute.org/gsea/index.jsp) introduced the Gene Set Enrichment Analysis (GSEA). From the MSigDB gene database, hallmark gene sets were obtained, and samples were categorized into high and low groups based on GGT5’s median expression level. Subsequently, GSEA was executed using default weighted enrichment statistics, ranking genes through the Pearson method. Gene sets significantly enriched in the high group were identified as GGT5-positively associated gene sets, whereas those significantly enriched in the low group were recognized as GGT5-negatively correlated gene sets. A gene set with NES > 1 and FDR < 0.05 was deemed statistically significant.

### Cell line cultivation

We procured human gastric cancer cell lines (SGC7901, AGS, HGC-27, MKN-45, NCI-N87, SNU-1) and the normal mucosal cell line (GES-1) from the Cell Bank of the Chinese Academy of Sciences (Shanghai, China). Maintained routinely in RPMI-1640 (Gibco, USA) at 37 °C under a 5% CO2 atmosphere, the cells were supplemented with 10% fetal bovine serum (Gibco, USA), along with 1% streptomycin and penicillin (HyClone, Logan, UT, USA).

### Quantitative real-time PCR, western blot

For RNA extraction from gastric tissues or cells (GES-1, SGC7901, AGS, HGC-27, MKN-45, NCI-N87, SNU-1), TRIzol reagent (Invitrogen, USA) was utilized, followed by reverse transcription into cDNAs using the HiScriptQ Select RTSuperMix for qPCR (Vazyme, China) as per the manufacturer’s instructions. Real-time quantitative PCR was then performed using the ChamQ™ Universal SYBR qPCR Master Mix (Vazyme, China). Triplicate real-time PCRs were conducted, and changes in gene expression were normalized to GAPDH expression, calculated using the 2 − ΔΔCt method. The primers used were as follows: GAPDH: F’-GTCAAGGCTGAGAACGGGAA, GAPDH: R’-AAATGAGCCCCAGCCTTCTC, GGT5: F’-GTGAGTTTGGCCACTCCGTA, GGT5: R’-GAATGGACAGATGGCTGGCA.

Western blotting (WB) adhered to standard protocols. Total cellular or tissue protein was lysed with RIPA buffer supplemented with a protease inhibitor and phosphatase inhibitor cocktail. Protein content was quantified using the BCA Protein Assay Kit (ThermoFisher Scientific, West Palm Beach, FL). Equal amounts (50 μg) of proteins underwent electrophoresis on 10% or 15% SDS polyacrylamide gels and were subsequently transferred to NC membranes (Pall Corporation). After blocking with 5% skim milk for one hour, membranes were cropped and incubated overnight at 4 °C with the specified primary antibodies. Following TBST washes, blots were labeled with HRP-conjugated secondary antibodies (Santa Cruz Biotechnology) and then detected using the ECL kit (Pierce Biotech, Rockford, IL). ImageJ software (http://imagej.nih.gov/ij) was employed to analyze the band intensities of the WB. Antibodies utilized in this study included GGT5 (#12002–1-AP, 1:1000, Proteintech, China), N-cadherin (#13116, 1:1000, Cell Signaling Technology, USA), E-cadherin (#3195, 1:1000, Cell Signaling Technology, USA), Vimentin (#5741, 1:1000, Cell Signaling Technology, USA), ZEB1 (#3396, 1:1000, Cell Signaling Technology, USA), TWIST1 (#90445, 1:1000, Cell Signaling Technology, USA), GAPDH (#2118, 1:1000, Cell Signaling Technology, USA).

### Tissue microarray and immunohistochemical (IHC) staining

We procured a human tissue microarray (TMA) (cat no. HStmA180Su09) comprising matched gastric tumor and normal tissue samples from 90 cases from Shanghai Outdo Biotech Co., Ltd. (Shanghai, China). Table 1 displays comprehensive clinical characteristics, encompassing patient age, gender, tumor size, histologic differentiation, and TNM stage. All GC patients underwent radical surgery between December 2009 and June 2010, with subsequent follow-up until June 2016. Individuals subjected to chemotherapy or radiotherapy before surgery were excluded from the study.

**Table 1:** Relationship between GGT5 levels and clinicopathological parameters of GC patients

Clinicopathological parameters	Cases(N)	GGT5 expression level		χ^2^	*P value*
		Low	High		
Age at surgery (years)					
< 60		8	20		
≥ 60		31	31	3.607	0.058
Gender					
Male		28	41		
Female		11	10	0.913	0.339
Tumor location					
Cardia		10	8		
Other sites		9	22		
Antrum		20	21	4.172	0.124
Tumor size (cm)					
< 5		18	18		
≥ 5		21	33	1.086	0.297
Histological differentiation					
Well		17	12		
Moderate/poor		22	39	4.072	0.044
Neural/vascular invasion					
No		32	40		
Yes		7	11	0.181	0.671
Lymph node metastasis (n)					
< 3		19	19		
≥ 3		20	32	1.19	0.275
TNM stage					
I–II		19	17		
III–IV		20	34	2.179	0.14

Following the manufacturer’s guidelines, we conducted IHC staining on TMA sections. Slides were subjected to staining using a rabbit anti-GGT5 polyclonal antibody (1:200 dilution; cat. no. sc-373693; Santa Cruz Biotechnology, USA). Two experienced pathologists, in a double-blind fashion, assessed the Immunoreactive Score (IRS) of each section. The IRS score system, as previously outlined [19], involved multiplying the staining intensity (0, negative; 1, weak; 2, moderate; and 3, strong) by the percentage of positive cells, categorized into five gradations (0, negative; 1, < 10%; 2, 10%–50%; 3, 51%–80%; and 4, > 80%). Three different fields were assessed for each sample. The IRS score ranged from 0–12, where IRS ≤ 4 indicated low GGT5 expression and IRS > 4 indicated high GGT5 expression.

### Vector construction and cell transfection

Invitrogen synthesized one shRNA targeting GGT5 (GGT5-shRNA) and a nontargeting scrambled RNA (Ctrl-shRNA), both of which were cloned into the lentivirus vector pLKO.1. Lentivirus packaging utilized HEK293T cells. Co-transfection of pLKO.1-GGT5-shRNA and pLKO.1-Control-shRNA occurred with lentivirus packaging plasmids psPAX2 (Addgene plasmid, 12260) and pMD2.G (Addgene plasmid, 12259) using Lipofectamine® 2000 (Invitrogen, USA). Virus harvesting transpired at 48 h and 72 h post-transfection. Cells underwent stable transfection with pLKO.1-GGT5-shRNA, and negative control viruses were chosen through puromycin treatment (5 μg/mL, Solarbio, China). The shRNA sequences are outlined below: shGGT5-forward: 5'-CCGGTTGTAGAGACGCTCAAGTTTGCTCGAGCAAACTTGAGCGTCTCTACAATTTTTG-3', shGGT5-reverse: 5'-AATTCAAAAATTGTAGAGACGCTCAAGTTTGCTCGAGCAAACTTGAGCGTCTCTACAA-3'.

### Cell counting kit-8 (CCK-8) and colony formation assay

To gauge the impact of GGT5 on cellular proliferation, a cell viability assay was conducted. Ninety-six-well plates were seeded with 1500 cells per well, incubated overnight at 37 °C in a 5% CO2 environment. At 24, 48, 72, 96, and 120 h, 10 μL of Cell Counting Kit-8 (CCK-8) solution (Beyotime Institute of Biotechnology, Shanghai, China) was introduced to each well and incubated for 2 h. Absorbance was then assessed using a microplate reader (Thermo Fisher Scientific, Waltham, MA, USA) at 450 nm.

For a comprehensive evaluation of the prolonged effects of GGT5 on cellular viability, a colony formation assay was employed. Each cell group was seeded into 6-well plates at a density of 1 × 103 cells per well under a 37 °C, 5% CO2 atmosphere. The culture medium was renewed every 3 days, and after 14 days of incubation, colonies were fixed with 4% paraformaldehyde for 15 min, followed by staining with 0.1% crystal violet for 30 min at room temperature. Subsequently, the colonies’ numbers were scanned and quantified using ImageJ software. Each experiment was independently conducted in triplicate.

### Wound healing assay

Six-well plates were utilized to seed AGS and HGC-27 cells, which were cultured in a medium containing 10% FBS. Once the monolayer cells achieved 90–100% confluence, a vertical scratch wound was created at the plate’s base using a 200-µl pipette tip. Subsequently, PBS was employed to wash away the floating cells, and the remaining cells were cultured in serum-free medium. Photographs were captured at 0 h and 24 h post-scratching. The wound healing rate was quantified using the ImageJ software.

### Cell migration and invasion assays

To evaluate cell migration and invasion, 24-well plates equipped with 8 µm pore transwell chambers (Corning, Inc, USA) were employed. For migration assays, transfected cells underwent a 12-h starvation period, followed by suspension of 2 × 105 cells in serum-free RPMI-1640 medium and placement in the upper chamber. Concurrently, RPMI-1640 medium containing 20% FBS was introduced into the lower chamber as a chemoattractant. In invasion assays, the Matrigel matrix was diluted at a 1:9 ratio and applied to the upper side surface of the transwell chamber’s bottom membrane. Subsequently, cells were seeded in the chamber as described in the migration assay, along with the medium. After a 24-h incubation at 37℃, residual cells on the upper surface of the filter membrane were delicately removed with a cotton swab. The migrated cells on the lower surface of the filter membrane were fixed with 4% paraformaldehyde and stained with 1% crystal violet for 15 min separately. Microscopic images were captured, and cell numbers were quantified using ImageJ software.

### Statistical analysis

Statistical analyses were conducted using R 4.1.3 (R Foundation for Statistical Computing, Vienna, Austria) and GraphPad Prism 10 (GraphPad Software, La Jolla, CA, USA). For comparisons between two groups, the Student’s t-test was applied. Categorical comparisons utilized Pearson’s χ2 test and Fisher’s exact test. Survival analyses were performed using the Kaplan–Meier method. Univariate and multivariate survival analyses were carried out with the Cox proportional hazards regression model. Results represent a minimum of three independent experiments; significance levels were denoted as * *p* < 0.05, ** *p* < 0.01, *** *p* < 0.001.

## Results

### Elevated transcriptional levels of GGT5 in human GC tissues

**Fig. 1:**
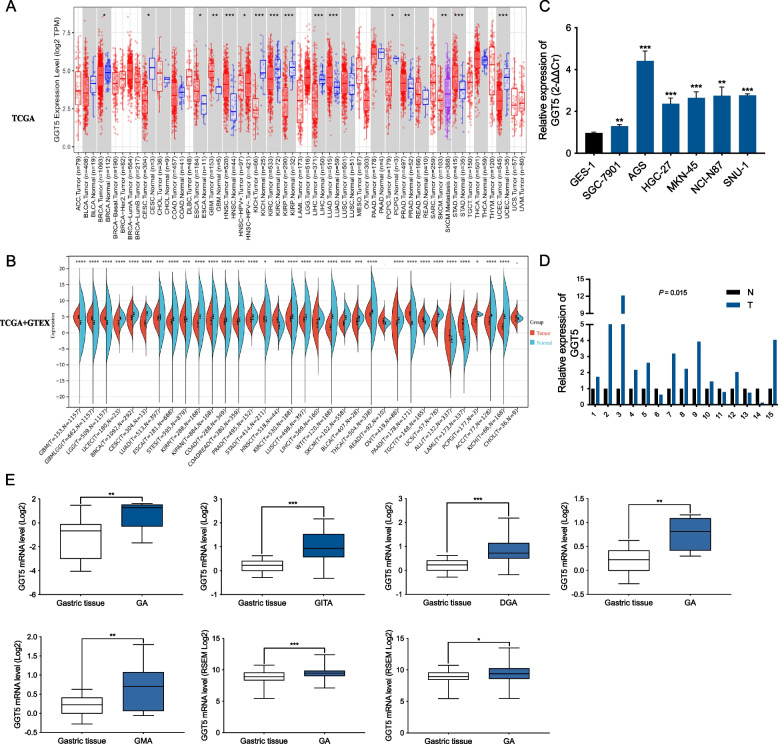
Transcriptional level of GGT5 were elevated in human gastric tissues. **A** Transcriptional levels of GGT5 in different types of cancers from TCGA database; **B** Transcriptional levels of GGT5 in different types of cancers from TCGA and GTEx database; **C** Relative expression levels of GGT5 in gastric cancer cells and normal gastric mucosal cells were determined by RT-qPCR; **D** Relative expression levels of GGT5 in gastric cancer tissues and parental normal tissues were determined by RT-qPCR; **E** Data from the TCGA and Oncomine databases were used to examine the differential expression levels of GGT5 mRNA between GC and normal gastric tissues. *p < 0.05, **p < 0.01 and ***p < 0.001

Our initial exploration encompassed the expression of GGT5 across diverse cancers, utilizing a comprehensive pan-cancer dataset derived from TCGA and GTEx databases (Genotype-Tissue Expression). TCGA results revealed a significant increase in the mRNA expression level of GGT5 in various cancers, including esophageal carcinoma (ESCA), glioblastoma multiforme (GBM), head and neck squamous cell carcinoma (HNSC), lung adenocarcinoma (LUAD), prostate adenocarcinoma (PRAD), and stomach adenocarcinoma (STAD) (all *p* < 0.05). Additionally, GGT5 exhibited heightened expression in HPV-HNSC- and SKCM (skin cutaneous melanoma) metastasis (*p* < 0.05). Conversely, GGT5 demonstrated a notable reduction in invasive breast carcinoma (BRCA), cervical squamous cell carcinoma, and endocervical adenocarcinoma (CESC), kidney chromophobe (KICH), kidney renal clear cell carcinoma (KIRC), kidney renal papillary cell carcinoma (KIRP), liver hepatocellular carcinoma (LIHC), pheochromocytoma and paraganglioma (PCPG), and uterine corpus endometrial carcinoma (UCEC) (all *p* < 0.05) (Fig. 1A). Subsequent cross-analysis with the GTEx database unveiled elevated GGT5 levels exclusively in GBM, HNSC, and STAD tumor tissues (*p* < 0.05), aligning with the downregulated GGT5 tumors observed in the TCGA database (Fig. 1B).

Furthermore, our investigation indicated higher GGT5 expression in gastric cancer cell lines compared to normal mucosal cell lines (*p* < 0.01) (Fig. 1C). Examining mRNA levels in 15 pairs of gastric cancer and parental normal tissues from our center revealed elevated GGT5 levels in 11 cancer tissues (*p* < 0.05) (Fig. 1D). Utilizing data from TCGA and Oncomine databases, we scrutinized the differential expression of GGT5 mRNA between gastric cancer (GC) and normal gastric tissues. The outcomes illustrated a substantial elevation of GGT5 in GC compared to normal gastric tissues (*p* < 0.05, Fig. 1E).

### Genetic alterations and mutation prognosis of GGT5

**Fig. 2:**
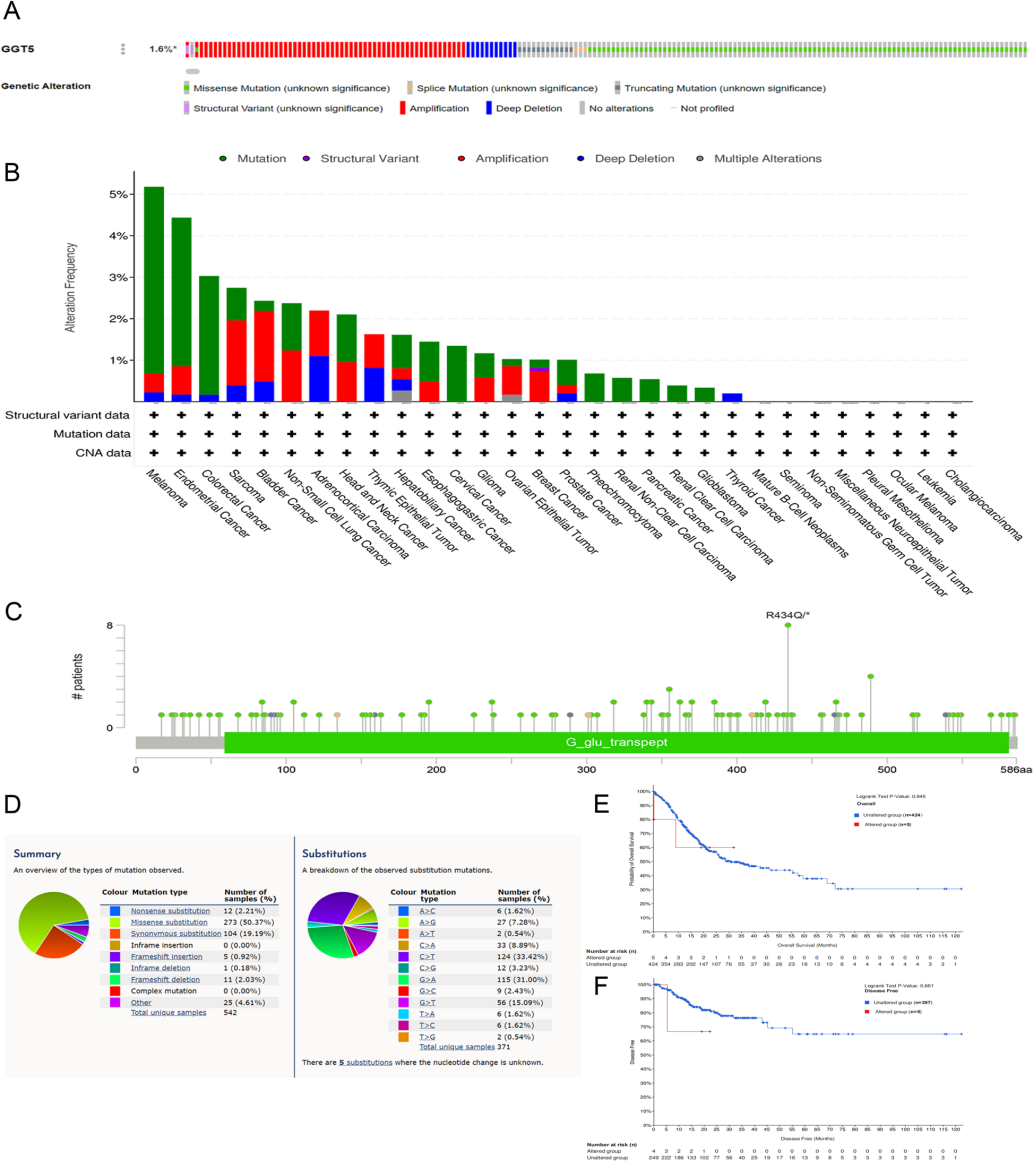
Genetic and epigenetic alterations of GGT5. **A** Genetic mutation in GGT5; **B** GGT5 mutation in pan-cancer; **C** Mutation sites of GGT5 in pan-cancer; **D** Mutation type of GGT5 in pan-cancer; **E**–**F** Correlation of GGT5 gene mutation with OS and DFS. *p < 0.05, **p < 0.01 and ***p < 0.001

We explored GGT5 gene mutations and alterations through cBioPortal. The alteration frequency was observed in patients with stomach adenocarcinoma (STAD) (1.6%), with “Missense Mutation” and “Amplification” being the most prevalent alterations (Figs. 2A-B). Additionally, we investigated specific mutation types and sites of GGT5 in cancer (Fig. 2C). The findings revealed that GGT5 alterations comprised nonsense substitution, missense substitution, synonymous substitutions, frameshift insertion, inframe deletion, and frameshift deletion, with missense substitution being the most prevalent (50.37%). Various nucleotide changes were noted for multiple mutations, with C > T and G > A being the most dominant (33.42%; 31.00%) (Fig. 2D). We also explored the impact of GGT5 gene mutations on survival using the cBioPortal database. Survival curves indicated that genetic mutations in GGT5 did not significantly influence overall survival (*p* = 0.945) or disease-free survival (*p* = 0.861) in STAD patients (Fig. 2E-F).

### Association between GGT5 expression and infiltration of immune cells

**Fig. 3:**
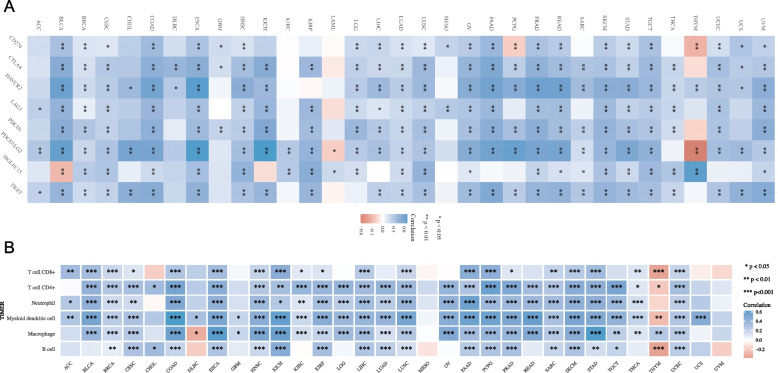
Relationship between GGT5 expression and immune cell infiltration. **A** Expression association between GGT5 and immune checkpoint-related genes; **B** The correlation coefficients of GGT5 expression with six immune cells: B cells, CD4 + T cells, CD8 + T cells, neutrophils, macrophages and dendritic cells. *p < 0.05, **p < 0.01 and ***p < 0.001

As the expression of immune checkpoint genes significantly influences immunotherapy efficacy, we utilized the TCGA database to investigate the expression correlation between GGT5 and immune checkpoint-associated genes: SIGLEC15, IDO1, CD274, HAVCR2, PDCD1, CTLA4, LAG3, and PDCD1LG2 across various cancers. The findings revealed a positive relationship between elevated GGT5 expression and immune checkpoint-related genes in most cancer types, particularly in STAD, excluding thymoma (THYM), PCPG, acute myeloid leukemia (LAML), KICH, and bladder urothelial carcinoma (BLCA) (Fig. 3A). These results suggest a potential interplay between GGT5 and immune checkpoint genes in shaping the tumor immune microenvironment.

Subsequently, we utilized the TIMER2 database to investigate the correlation between GGT5 expression and immune cell infiltration levels in pan-cancer. The heat map illustrated correlation coefficients of GGT5 expression with six immune cell types (B cells, CD4 + T cells, CD8 + T cells, neutrophils, macrophages, and dendritic cells) collected from the TIMER2 database (Fig. 3B). Notably, the strongest positive correlation between GGT5 and immune cell infiltration was observed in STAD, while the most negative correlation was found in THYM. Myeloid dendritic cells exhibited significant coefficients across various malignancies, including BLCA, COAD, THCA, UCS, and UVM. These findings suggest a close association between GGT5 expression and the extent of immune cell infiltration.

### Elevated GGT5 levels in GC and their correlation with unfavorable prognosis in patients with GC

**Fig. 4:**
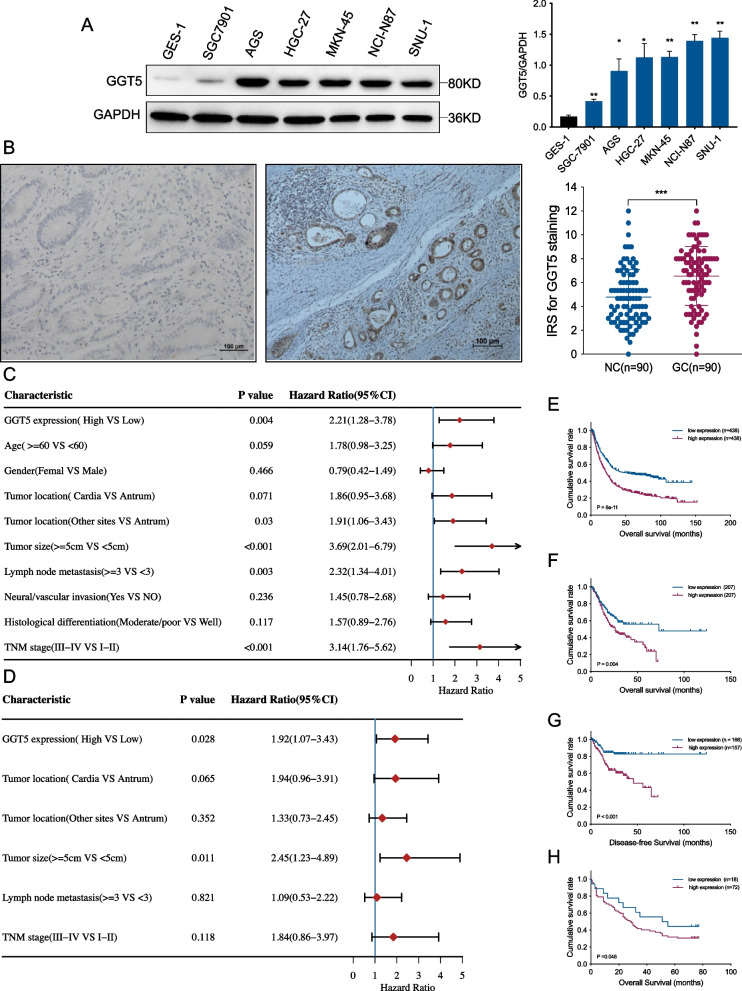
GGT5 was increased in GC and associated with poor prognosis of GC patients. **A** Western blotting showed the expression of GGT5 in human gastric mucosal epithelial cell line and gastric cancer cell lines; **B** Relative expression levels of GGT5 in gastric cancer tissues and neighboring noncancerous tissues were detected by IHC (*p* < 0.001, *n* = 90); **C** A forest map of the results of the univariate analysis; **D** A forest map of the results of the multivariate analysis; **E**–**G** Kaplan–Meier survival curves for OS and DFS based on GGT5 expression from TCGA and the Kaplan–Meier plotter database; **H** Kaplan–Meier survival curves for OS based on GGT5 expression from IHC results. *p < 0.05, **p < 0.01 and ***p < 0.001

To further investigate GGT5 expression in gastric cancer, we utilized Western blotting (WB) and immunohistochemistry (IHC) to evaluate GGT5 protein levels in gastric cancer cell lines and 90 cases of gastric cancer along with their corresponding adjacent normal tissue samples. GGT5 protein levels in six gastric cancer cell lines and a normal human gastric epithelial cell line (GES-1) were assessed using WB, revealing elevated GGT5 protein levels in all gastric cancer cell lines compared to GES-1 (Fig. 4A). IHC assays were conducted to assess GGT5 protein levels in the 90 cases of gastric cancer and their paired adjacent normal tissues. The immunoreactive score (IRS) of GGT5 in tumors was significantly higher than that in adjacent noncancerous gastric tissues (*p* < 0.001) (Fig. 4B). Associations between GGT5 protein expression and clinicopathological parameters (age, gender, tumor location, tumor size, histological differentiation, neural/vascular invasion, lymph node metastasis, and TNM stage) in patients with gastric cancer were explored. The dichotomized expression level of GGT5 protein (low versus high) was notably associated with histological differentiation in patients with gastric cancer (*p* < 0.05; Table 1).

Univariate and multivariate analyses were conducted to identify risk factors for gastric cancer. Univariate regression analysis revealed factors influencing overall survival (OS), including GGT5 expression, tumor location (Other sites vs. Antrum), tumor size, lymph node metastasis, and TNM stage (all *p* < 0.05) (Fig. 4C). Additionally, multivariate analysis demonstrated that GGT5 expression and tumor size were independent risk factors for gastric cancer progression (all *p* < 0.05) (Fig. 4D).

The prognostic significance of GGT5 expression levels in gastric cancer patients was also explored. Through data mining in the TCGA database and the Kaplan–Meier plotter (http://kmplot.com/analysis/index.php?p=service), we observed significantly lower overall survival (OS) and disease-free survival (DFS) among gastric cancer patients with high GGT5 expression compared to those with low expression, using the median as the cutoff value (all *p* < 0.05, Fig. 4E-G). As GGT5 is a protein-coding gene, to validate the predictive results, we also analyzed the IHC staining results, revealing significantly lower overall survival (OS) among gastric cancer patients with high GGT5 expression protein compared to those with low expression (*p* = 0.046; Fig. 4H).

### GGT5 enhances the proliferation, migration, and invasion of GC cells

**Fig. 5:**
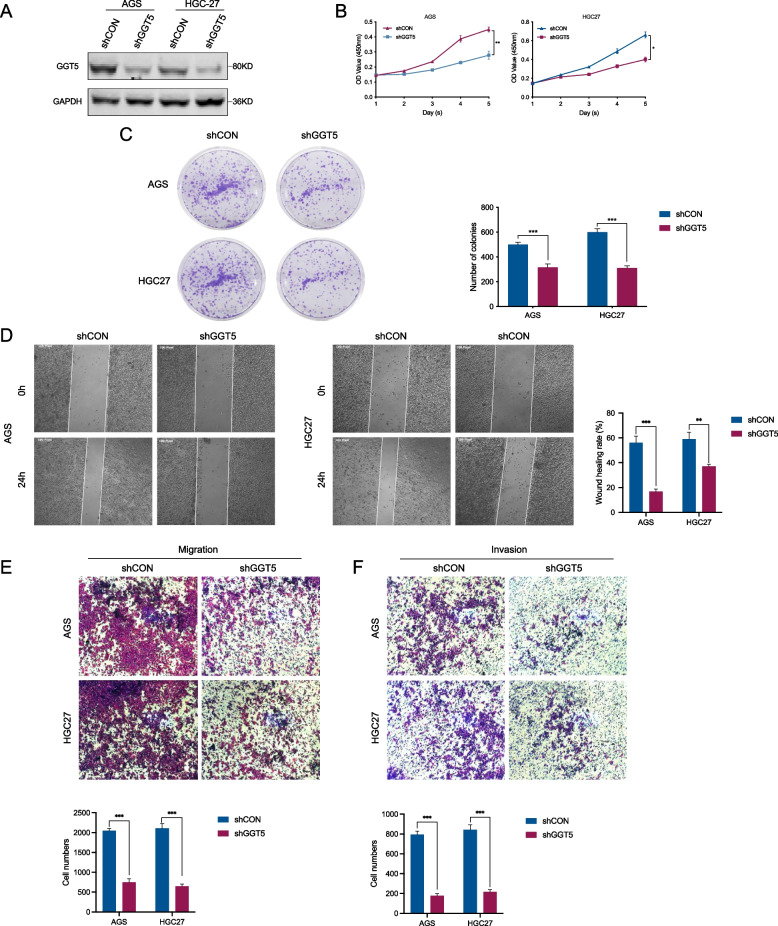
GGT5 promotes the proliferation, migration, and invasion of GC cells. **A** AGS and HGC27 cells were transfected with GGT5-shRNAs; **B** Cell Counting Kit-8 was used to detect the proliferation of AGS and HGC27 cells after GGT5 knockdown; **C** The wound healing assay was used to detect the migration of AGS and HGC27 cells after GGT5 knockdown; **E**–**F** Transwell assays were used to detect the migration and invasion of AGS and HGC27 cells after GGT5 knockdown. *p < 0.05, **p < 0.01 and ***p < 0.001

To validate the impact of GGT5 in GC cells, we introduced GGT5-shRNA into AGS and HGC27 cells to downregulate GGT5 expression. The effectiveness of suppression was verified through WB analysis (Fig. 5A). To ascertain GGT5’s influence on AGS and HGC-27 cell proliferation, CCK8 and colony formation assays were conducted. In CCK8 experiments, the proliferation rate of GGT5-shRNA-treated AGS and HGC-27 cells markedly decreased compared to the shCtrl group (Fig. 5B). Similarly, the number of colonies formed by GGT5-shRNA-treated cells significantly diminished compared to the shCtrl group (Fig. 5C).

For an in-depth exploration of GGT5’s impact on GC cell migration and invasion, wound healing and transwell migration/invasion assays were executed. Results from the wound healing and transwell migration assays demonstrated a substantial decrease in migration in the GGT5-shRNA group compared to the shCtrl group (Fig. 5D-E). Furthermore, the transwell invasion assay with Matrigel exhibited reduced invasive capability in the GGT5-shRNA group (Fig. 5F).

### GGT5 potentially regulates epithelial-mesenchymal transition (EMT) processes in gastric cancer

**Fig. 6:**
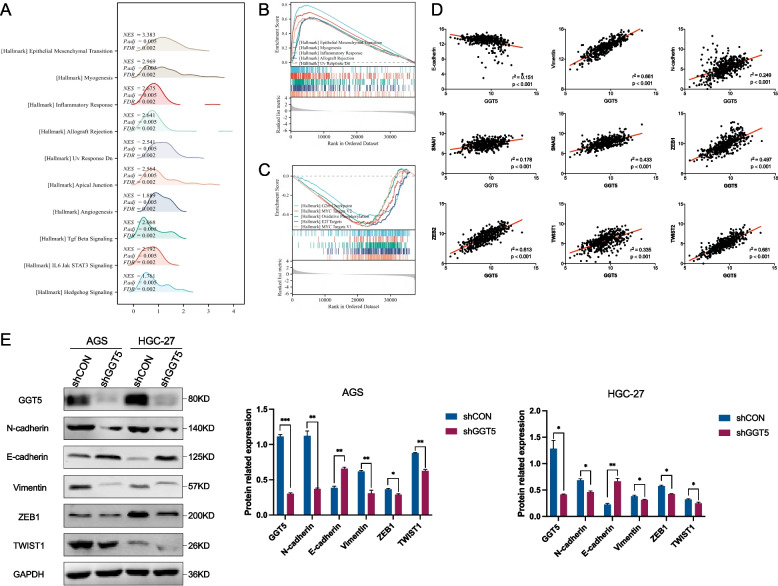
GGT5 may function by modulating epithelial‑mesenchymal transition. **A**-**C** GSEA enrichment plots indicated that GGT5 expression was positively correlated with the EMT gene signature; **D** GGT5 was significantly correlated with several genes associated with EMT; **C** Western blotting analysis was used to validate the difference in the expression of epithelial and mesenchymal markers in GC cells infected with GGT5-shRNAs or scramble. *p < 0.05, **p < 0.01 and ***p < 0.001

To delineate the potential mechanism underlying GGT5-mediated tumor progression, this study utilized RNA-seq data from TCGA to perform gene set enrichment analysis (GSEA) [20, 21]. The findings indicated a positive correlation between GGT5 expression and genes associated with processes such as EMT, Myogenesis, Inflammatory Response, Allograft Rejection, Uv Response Dn, etc. (all *p* < 0.05) (Fig. 6A-B). Conversely, GGT5 expression exhibited a negative correlation with genes involved in G2m Checkpoint, Myc Targets V2, Oxidative Phosphorylation, E2f Targets, Myc Targets V1, etc. (all *p* < 0.05) (Fig. 6C). Moreover, Fig. 6D illustrates a positive correlation between GGT5 expression and Vimentin, N-cadherin, snail family transcriptional repressors (Snai1, Snai2), TWIST1, TWIST2, zinc finger E-box binding homeoboxes (ZEB1, ZEB2), while showing a negative correlation with E-cadherin. Additionally, GGT5 silencing in GC cells led to increased protein expression levels of the epithelial marker E-cadherin, coupled with decreased levels of mesenchymal markers N-cadherin, Vimentin, TWIST1, ZEB1 (Fig. 6E). These findings collectively suggest that GGT5 actively contributes to tumor aggressiveness through EMT in GC.

## Discussion

Despite considerable advancements in diagnosis, intervention, and therapy, the prognosis for GC patients remains suboptimal, with a poor survival rate [22, 23]. Comprehensive therapy often proves ineffective in later-stage patients, emphasizing the need for a deeper understanding of disease progression mechanisms and the identification of potential therapeutic targets. Molecular biomarkers, serving as prognostic and diagnostic features, are increasingly employed in clinical practice, greatly aiding patient classification, disease state monitoring, and personalized treatment planning [24, 25]. Recent investigations have unveiled a correlation between elevated serum GGT (γ-glutamyl transferase) levels and the prognosis of various digestive tumors. Lyu et al. demonstrated that the combined examination of CA19-9/GGT, compared to CA19-9 levels alone, provided a more precise evaluation of postoperative tumor recurrence and long-term prognosis in PHC (pancreatic head carcinoma) patients [26]. In patients with CRC (colorectal cancer) and hepatic metastases, high baseline serum alkaline phosphatase (AKP) and GGT were associated with worse overall survival. The measurement of serum AKP or GGT changes before and after the first treatment cycle served as a convenient, rapid, and economical means to early predict antitumor treatment efficacy [27]. Another study conducted multivariate analysis, identifying preoperative serum GGT as an independent predictor of OS, CSS (cancer-specific survival), and DFS in bladder cancer (BC) patients following radical cystectomy, suggesting its inclusion in prognostic models [28]. The aforementioned studies primarily focused on serum GGT5 levels in predicting tumor prognosis, with several exploring the expression significance of GGT5 in tumor tissues. Li et al. revealed high GGT5 expression in CAFs of lung adenocarcinoma, where elevated GGT5 levels in CAFs contributed to cancer cell survival and drug resistance, predicting unfavorable survival in lung adenocarcinoma patients [13]. Additionally, a study on B-cell malignancy indicated that GGT5 was overexpressed by follicular dendritic cells, disrupting P2RY8’s ability to promote B cell confinement to germinal centers [29].

Moreover, GGT5, a conventional biochemical indicator, is extensively employed for assessing the severity of GC. In the context of overall survival in GC patients, four genes associated with antioxidants (CHAC1, GGT5, GPX8, and PXDN) demonstrated significance. However, only CHAC1 (HR = 0.803, *P* < 0.05) and GPX8 (HR = 1.358, *P* < 0.05) emerged as independent factors in one study [30]. Another investigation identified 13 potential prognostic differentially expressed MRGs (metabolism-related genes), including GGT5, constructing a valuable metabolic model for predicting GC prognosis [14]. In a subsequent study, Ye and colleagues established a prognostic prediction model containing 13 MRGs and explored the association between metabolism and the immune microenvironment in STAD [15]. Wang reported that the high-expression GGT5 group exhibited elevated concentrations of M2 macrophages, T cell regulators, and monocytes, while the low-expression GGT5 group had higher plasma cell and M1 macrophage contents. Higher PD1 and CTLA4 expression levels were observed in the high-expression GGT5 group, suggesting its potential effectiveness in immunotherapy [31]. Concurrently, another group found a positive correlation between GGT5 expression and the infiltration of natural killer cells, macrophages, and dendritic cells, with a negative correlation with Th17 infiltration. Furthermore, GGT5 demonstrated positive co-expression with immune-related genes and immune checkpoint genes, indicating its potential as an immunological therapeutic target for GC [32].

While the correlation between GGT5 and GC prognosis has been previously explored, the specific role of GGT5 remains unclear. In our investigation, pan-cancer analysis bioinformatics indicated heightened GGT5 expression in numerous tumor tissues, including GC tissues, compared to normal tissues. This observation was further substantiated by IHC results obtained from clinical samples at our center, aligning with findings from online databases. Furthermore, elevated GGT5 levels in GC tissue were predictive of unfavorable DFS and OS rates, consistent with conclusions from other studies [14, 15, 31]. Although prior research relied on TCGA or GEO databases, our multivariate analyses of clinical samples underscored the prognostic relevance of GGT5 in GC. Despite this, its function and underlying mechanisms remain unclear, warranting in-depth exploration of the intrinsic connections between GGT5 and GC. To address this gap, we suppressed GGT5 expression in GC cell lines, observing significant reductions in proliferation, migration, and invasion capacities. These outcomes suggest that targeting GGT5 could emerge as a viable strategy for GC treatment. In delving into the biological function of GGT5 in gastric cancer, GSEA analysis revealed potential significant enrichment pathways in the high-expression GGT5 group, particularly implicating involvement in EMT. Subsequent correlation analyses between GGT5 and EMT-related genes unveiled a positive correlation with E-cadherin and a negative correlation with N-cadherin, Vimentin, etc. These findings imply that GGT5 may foster the growth and metastasis of gastric cancer cells through the EMT pathway, contributing to the poor survival of gastric cancer patients. However, a precise regulatory mechanism necessitates further experimental exploration.

Several limitations persist in our study. Initially, GGT5, identified as a novel serum biomarker in PHC, CRC, and BC, serves as a secretory protein in serum. Therefore, assessing its serum concentration in GC patients may hold clinical significance. Secondly, to validate the in vitro findings obtained thus far, additional in vivo experiments are imperative. Third, the expression level of GGT5 in each stage of gastric cancer was not provided in detail in our study. However, cancer cell transcriptome changes with disease progression, as Barik etc. reported earlier: autoimmune liver diseases (AILD) often lead to transformation of the liver tissues into hepatocellular carcinoma (HCC), most of the regulatory interactions were operative during early (F0–F1) and intermediate fibrotic stages (F2–F3), while the extent of activity in the regulatory network considerably diminished at late stage of fibrosis/cirrhosis (F4) [33]. This will be the focus of our subsequent research. Fourth, As mutation-induced genomic instability, chromosomal deletions and rearrangements may cause the formation of tumor [34], so we explored the mutations of GGT5 in GC. However, although we have explored GGT5 gene mutations and alterations through bioinformatic tools, but which mutation is responsible for enhanced tumorigenic properties still needs to be explored in the future. Fifth, the exact mechanism by which GGT5 interplays during the EMT process remains incompletely understood. These aspects are pivotal and will be a primary focus in our future investigations.

## Conclusion

In summary, relying on numerous reputable databases, clinical specimens, and experimental substantiation, this study recognizes GGT5 as an innovative prognostic indicator. It proves valuable in forecasting the prognosis and immune response in gastric cancer patients. GGT5 is implicated in expediting tumor advancement by enhancing the proliferation and migration of GC cells through the modulation of EMT-associated pathways. Hence, this investigation underscores the imperative need for future research on the role of GGT5 in the tumorigenesis of GC.

### Supplementary Information

**Supplementary Material 1. **

## Data Availability

The data presented in this study are available on reasonable request from the corresponding author, Dr. Jun Ren.
