# Supplementary figures and images for: GGT5 facilitates migration and invasion through the induction of epithelial–mesenchymal transformation in gastric cancer
Source: BMC Med Genomics. 2024 Apr 5;17:82. doi: 10.1186/s12920-024-01856-0 (PMC10998378; doi:10.1186/s12920-024-01856-0)

figure 4A

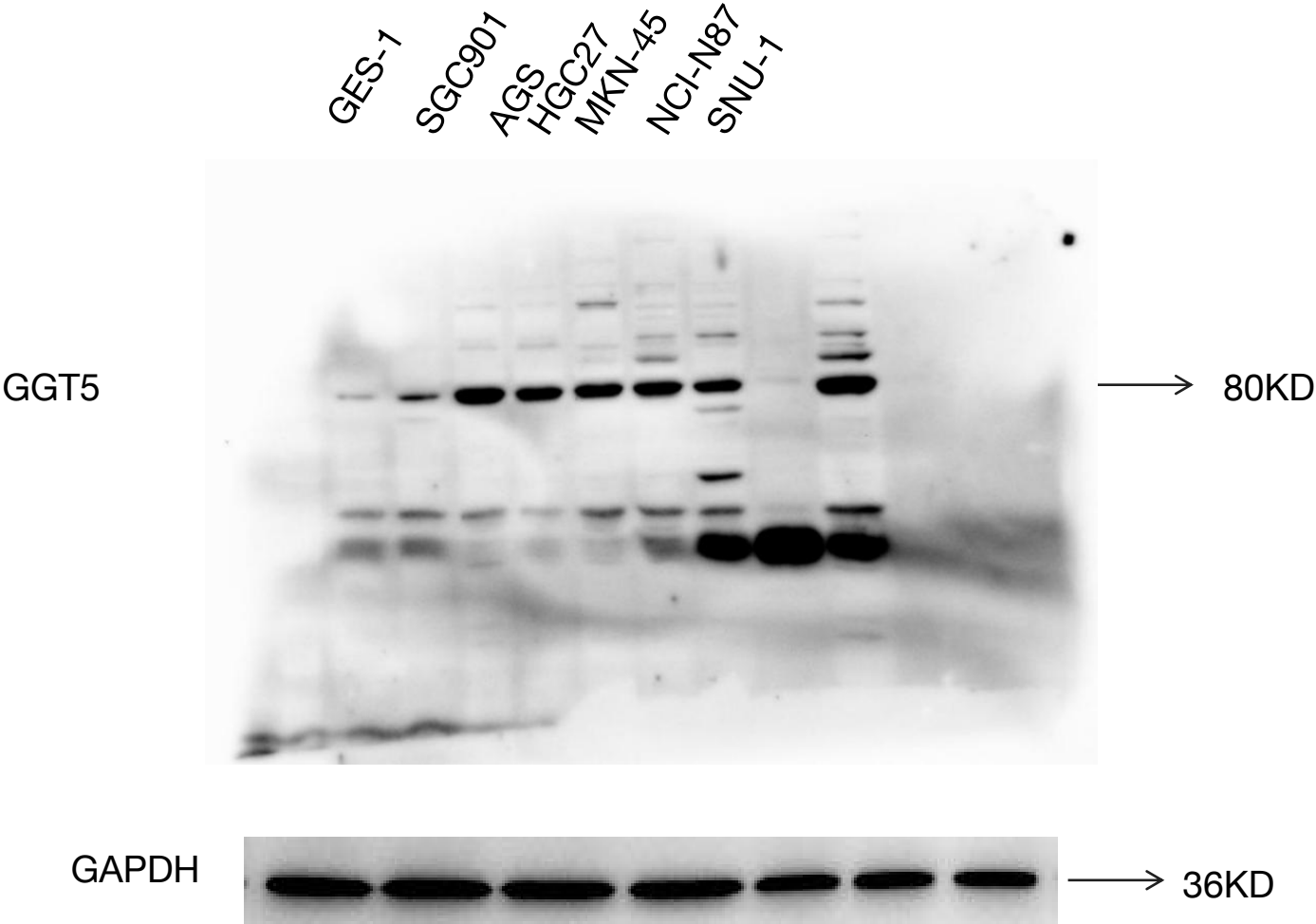

figure 5A

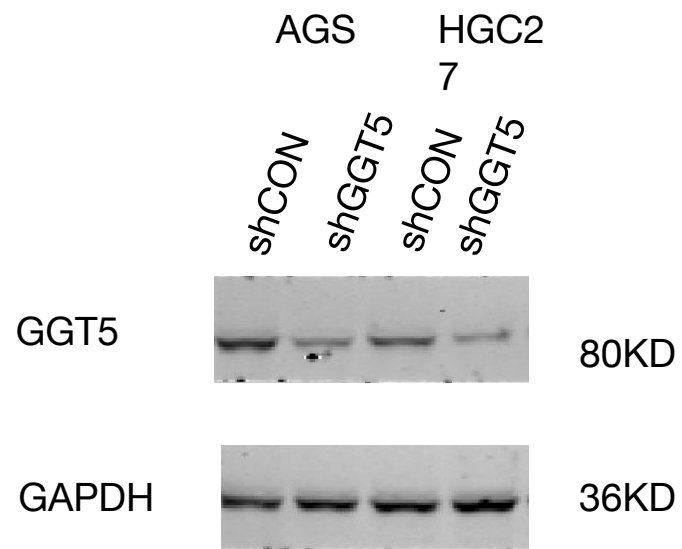

figure 6E

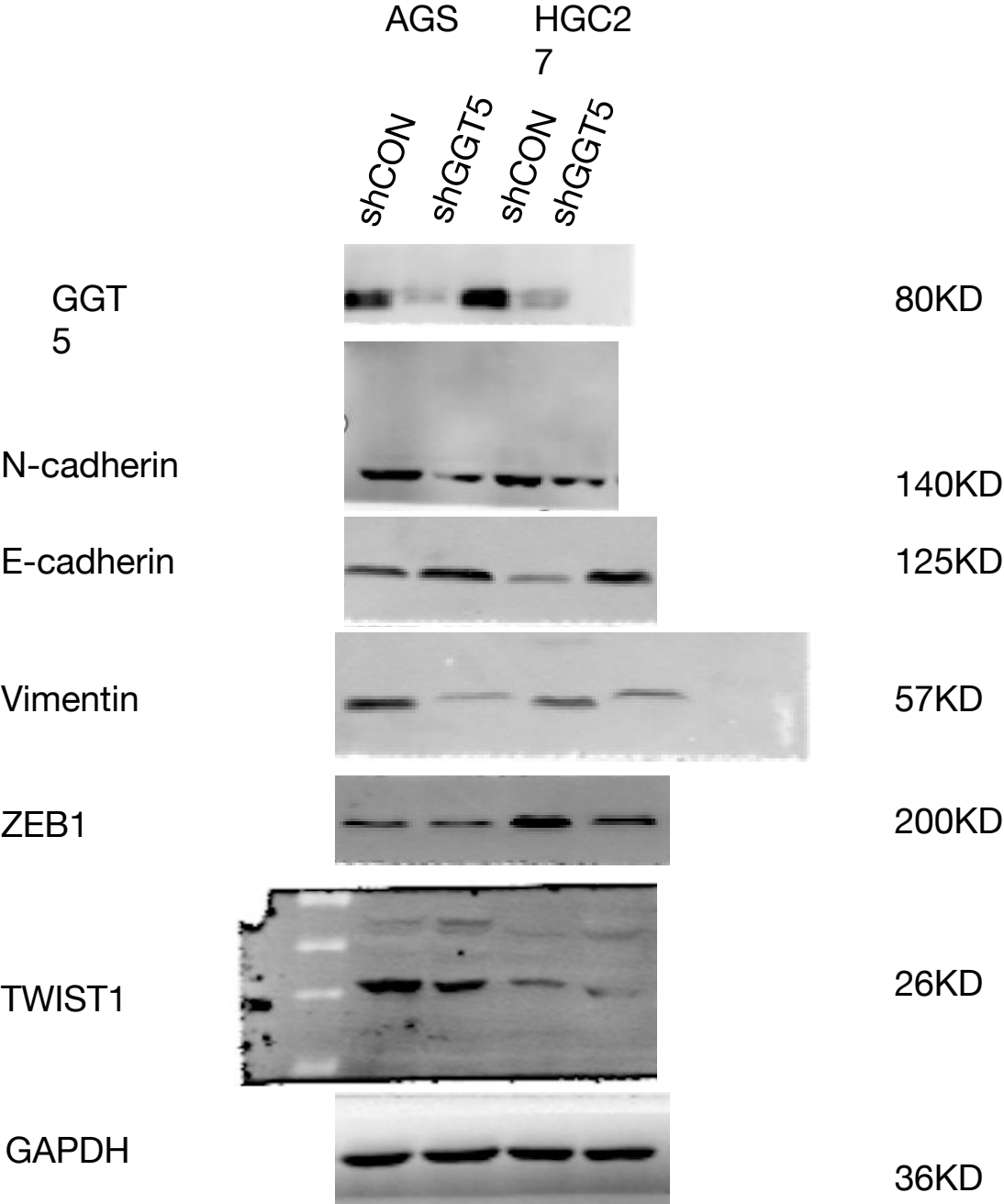

Supplement: Supplementary file 1 — Supplementary Material 1. [file 12920_2024_1856_MOESM1_ESM.pdf]
